# Gut Microbiota and Atherosclerosis: Integrative Multi-Omics and Mechanistic Insights

**DOI:** 10.1007/s11883-025-01371-2

**Published:** 2025-12-18

**Authors:** Jiahuan Helen He, Hanwen Wang, Eric Qiu, Qibin Qi, Zheng Wang

**Affiliations:** 1 Department of Epidemiology, Johns Hopkins Bloomberg School of Public Health, Baltimore, MD 21251 USA; 2 Department of Biomedical Engineering, Johns Hopkins Whiting School of Engineering, Baltimore, 21251 MD USA; 3 Department of Epidemiology and Population Health, Albert Einstein College of Medicine, 1300 Morris Park Avenue, Bronx, NY 10461 USA; 4 Department of Nutrition, Harvard T.H. Chan School of Public Health, Boston, MA 02108 USA

**Keywords:** Gut microbiota, Microbial metabolites, Atherosclerosis, Cardiovascular disease

## Abstract

**Purpose of Review:**

This review synthesizes and discusses evidence from metagenomics, metabolomics, and proteomics on gut microbiome alterations in atherosclerotic cardiovascular disease (ACVD), with carotid atherosclerosis (CAS) serving as an example.

**Recent Findings:**

Evidence on gut microbial α-diversity and β-diversity was mixed and differs by disease status. Pro-inflammatory/pathogenic gut bacterial taxa (e.g., *Escherichia coli*, *Klebsiella spp.*, *Streptococcus spp.*, and *Ruminococcus gnavus*) were often enriched in patients with ACVD or CAS, whereas short-chain fatty acid (SCFA) producers (e.g., *Faecalibacterium prausnitzii*, *Roseburia spp.*, *Bacteroides spp.*, and *Eubacterium eligens*) were depleted. Targeted and untargeted metabolomics implicated multiple microbial-derived metabolites in relation to ACVD and CAS, including trimethylamine N-oxide, short-chain fatty acids, bile acids, lipopolysaccharides, phenylacetylglutamine, indole-3-propionate and imidazole propionate.

**Summary:**

Gut dysbiosis contributes to ACVD or CAS possibly via metabolite-mediated effects on endothelial function, inflammation, and lipid metabolism. Future research prioritizing longitudinal and interventional studies integrating microbial metagenomics with host multi-omics are needed to elucidate causal pathways and identify clinically actionable targets.

## Introduction

Atherosclerotic cardiovascular disease (ACVD) remains the leading cause of global morbidity and mortality. In 2023, ACVD accounted for a substantial global health burden, with ischemic heart disease and ischemic stroke representing the two most common causes of cardiovascular death, causing approximately 8.9 million and 3.3 million deaths, respectively [1].

Carotid atherosclerosis (CAS), the buildup of plaque in the carotid arteries that transport blood to the head and brain, is a manifestation of systemic ACVD [2]. CAS is usually assessed by non-invasive carotid ultrasound imaging including the measurement of carotid intima-media thickness and the detection and quantification of carotid arterial plaque (presence, thickness, area, or volume) [3]. In 2020, the prevalence of increased carotid intima-media thickness and presence of carotid plaque was around 28% and 21%, respectively [4]. CAS can contribute directly to ischemic stroke when a carotid plaque ruptures, and the plaque fragments or thrombi can embolize into cerebral blood vessels, leading to ischemic stroke or transient ischemic attack [2].

The pathophysiology of CAS involves endothelial dysfunction, vascular smooth muscle dysregulation, abnormal lipid accumulation, and chronic vascular inflammation [2]. Traditional risk factors such as older age, dyslipidemia, high blood pressure, treated for hypertension, diabetes, obesity, and smoking only partially explain the variability in plaque burden [5, 6]. Prior research has shown that these factors explain less than 20% of the variability in carotid plaque area, and even after incorporating socioeconomic determinants, the proportion of variability explained improved only marginally [7]. This suggests that additional non-traditional factors may play a role in determining individual susceptibility to atherosclerosis.

Recently, gut microbiota has emerged as another potential player in atherogenesis [8, 9]. Gut dysbiosis, or imbalance in gut microbial composition, has been linked to the generation of pro-atherogenic metabolites such as trimethylamine N-oxide (TMAO), as well as the reduction of protective short-chain fatty acids (SCFAs) [8, 9].

This review focuses on ACVD, using CAS as an example, given the direct clinical relevance of CAS to stroke, where it accounts for 15%-20% of the ischemic stroke cases [10], as well as its utility to predict the risk of cardiovascular disease [11]. We summarize current evidence on gut microbiota alterations in patients with subclinical or clinical ACVD, discuss the potential mechanism, and highlight key research gaps and future directions for leveraging gut microbiota to improve CAS risk assessment and therapy.

A systematic search was performed in PubMed covering studies published from January 2010 through October 2025. Search terms included “gut microbiota”, “microbiome”, “atherosclerosis”, “carotid atherosclerosis’, and “stroke”. We identified research articles through a manual review process of the selected publications.

## Alterations of Gut Microbiota in ACVD Patients

In healthy adults, *Bacteroidetes* and *Firmicutes* are the major bacterial phyla in the gut accounting for more than 90% of the total bacterial taxa, followed by a smaller portion of phyla *Proteobacteria* and *Actinobacteria* [12]. Accumulating evidence suggests that patients with CAS or broader AVCD have gut microbial communities that differ from those of healthy individuals. These differences are not only observed in overall microbial diversity but also in the relative abundance of specific taxa that may be pro- or anti-atherogenic.

### α-Diversity

Findings on microbial richness and evenness in ACVD, including CAS, are inconsistent. Most studies reported no significant differences in microbial richness or evenness between individuals with atherosclerotic conditions and controls. However, a few studies such as Haak et al. (2020) [13] observed a reduction in α-diversity among patients with stroke (primarily ischemic stroke), implying a loss of microbial complexity in advanced cerebrovascular disease. Some evidence also indicates that specific cardiometabolic or clinical factors may correlate with α-diversity. For instance, Li R.J. et al. (2021) [14] reported that α-diversity was positively associated with blood pressure and follicle-stimulating hormone but negatively correlated with uric acid and liver enzyme gamma-glutamyl transferase (GGT) (Table 1).

**Table 1:** Overview of gut microbiome alterations in relation to atherosclerosis

Author (Year)	Location	Sample Size	Outcome	Study design	Methods	Increased Relative Abundance in Group with clinical/subclinical/risk factors of atherosclerosis	Decreased Relative Abundance in Group with clinical/subclinical/risk factors of atherosclerosis	Other Findings (Diversity, Metabolomics, Transcriptomics, Associations)
Chen et al., 2021 [15]	China	31 CAS, 51 controls	CAS	Case–control	Metagenomic shotgun sequencing	*Bacteroides eggerthii*, *Escherichia coli*, *Klebsiella pneumoniae*	*Parabacteroides* unclassified, *Prevotella copri*, *Bacteroides spp. 3_1_19*, *Haemophilus parainfluenzae*	No significant difference in α- or β-diversity observed between groups
Ji et al., 2021 [16]	China	32 CAS, 32 controls	CAS	Case–control	16S rDNA sequencing	*Acidaminococcus*, *Christensenella*, *Lactobacillus*	*Anaerostipes*, *Fusobacterium*, *Gemella*, *Parvimonas*, *Romboutsia*, *Clostridium XVIII/XIVa/XIVb*	Increased phenylacetylglutamine (PAGln); Increased FABP4 gene expression; FABP4 was positively correlated with *Acidaminococcus* and PAGln; FABP4 was negatively correlated with ethanolamine No significant difference in α-diversity, but β-diversity was significantly different between groups
Jie et al., 2017 [17]	China	218 ACVD, 187 controls	ACVD	Case–control	Metagenomic shotgun sequencing	*Escherichia coli*, *Klebsiella spp.*, *Enterobacter aerogenes*, *Streptococcus spp.*, *Lactobacillus salivarius*, *Solobacterium moorei*, *Atopobium parvulum*, *Ruminococcus gnavus*, *Eggerthella lenta*	*Roseburia intestinalis*, *Faecalibacterium prausnitzii*, *Bacteroides spp.*, *Prevotella copri*, *Alistipes shahii*	*Streptococcus spp.* negatively correlated with *Bacteroides spp.*; *Klebsiella oxytoca* correlated with aspartate aminotransferase (AST), creatine kinase MB; *Faecalibacterium prausnitzii* inversely correlated with uric acid No significant difference in α- or β-diversity observed between groups
Huang et al., 2023 [18]	Southern China	45 at risk of stroke, 15 controls	Stroke	Case–control	16S rRNA sequencing	*Anaerostipes*, *Clostridium XIVb*, *Flavonifractor*	–	No significant difference in α-diversity, but β-diversity was significantly different between groups
Karlsson et al., 2012 [19]	Sweden	12 CAS, 13 controls	CAS	Case–control	Metagenomic shotgun sequencing	*Collinsella*	*Roseburia*, *Eubacterium*	–
Szabo et al., 2021 [20]	Hungary	14 monozygotic twins with discordant carotid intima-media thickness	Carotid intima-media thickness,	Twin study	16S rRNA sequencing	*Firmicutes*, higher Firmicutes/Bacteroidetes ratio	*Prevotellaceae*	No α-diversity difference between groups; Higher risk of carotid intima-media thickness in high *Firmicutes* group
Zhu et al., 2022 [21]	Rural China	569 older adults with asymptomatic subclinical atherosclerosis	Asymptomatic subclinical atherosclerosis	Cross-sectional	Metagenomic shotgun sequencing	*Libanicoccus*	*Faecalicatena*	Healthier lifestyle led to lowered risk of CAS via *Alistipes*, *Oligella*, *Prevotella*; Blood chemistry, arterial stiffness, bone mass density, and carotid ultrasonographic measurements were predictors of phylum-level α-diversity; Heart function, disease status, demographic data, socio-economic status, and arterial stiffness were found to be strongly associated with β-diversity
Baragetti et al., 2021 [22]	Northern Milan	144 with subclinical CAS, 201 controls	Subclinical CAS	Cross-sectional	16S rRNA sequencing and metagenomic shotgun sequencing	*Escherichia*, *Shigella*, *Streptococcus* (*S. salivarius*, *S. parasanguinis*, *S. anginosus*), *Oscillospira*	*Bacteroides* (*B. uniformis*, *B. thetaiotaomicron*)	No significant difference in α-diversity; β-diversity was significantly different between groups
Stø et al., 2022 [23]	Norway	43 CAS, 38 controls	CAS	Case–control	16S rRNA sequencing	*Anaerotruncus*, *Ruminococcus gnavus*, *Lachnospiraceae CAG_56*	*Lachnospiraceae UCG_003*, *Eubacterium eligens*, *Coprococcus*	Mixed pattern among butyrate-producing taxa; No difference in top 10 genera between the two groups; No significant difference in α- or β-diversity observed between groups
Li R.J. et al., 2021 [14]	China	102 treatment-naïve CAS + metabolic dysfunctions; 36 treatment-naïve metabolic dysfunctions	CAS	Case–control	Metagenomic shotgun sequencing	*Clostridium bolteae*, *Tyzzerella nexilis*, *Ruminococcus gnavus*, *Blautia hansenii*, *Atopobium parvulum*, *Solobacterium moorei*	*Eubacterium eligens*, *Faecalibacterium prausnitzii*, *Ruminococcus lactaris*	Positive correlation between higher levels of gamma-glutamyl transferase (GGT), alanine aminotransferase (ALT), total bilirubin with pathogenic taxa; *E. eligens* inversely correlated with liver dysfunction markers; Blood pressure and follicle-stimulating hormone were positively correlated with α-diversity, while uric acid and GGT were negatively correlated with α-diversity No significant difference in α-diversity between groups
Li H. et al., 2020 [24]	China	79 cerebral infarction, 98 controls	Cerebral infarction	Case–control	16S rRNA sequencing	*Proteobacteria*, *Actinobacteria*	*Firmicutes*, *Bacteroidetes*	No significant difference in α- or β-diversity observed between groups
Haak et al., 2020 [13]	Netherlands	349 stroke (287 ischemic, 37 hemorrhagic), 51 controls	Stroke	Case–control	16S rRNA sequencing	*Escherichia/Shigella*, *Peptoniphilus*, *Ezakiella*, *Enterococcus*	*Blautia*, *Subdoligranulum*, *Bacteroides*	Decreased α- and β-diversity in patients with stroke; Controls and transient ischemic attack without stroke had higher SCFA-producing taxa
Tan et al., 2020 [25]	China	140 stroke, 92 controls	Stroke	Case–control	16S rRNA sequencing	*Lactobacillaceae*, *Akkermansia*, *Enterobacteriaceae*, *Porphyromonadaceae*	*Roseburia*, *Bacteroides*, *Lachnospiraceae*, *Faecalibacterium*, *Blautia*, *Anaerostipes*	Higher stroke severity was associated with fewer SCFA producers, more pro-inflammatory taxa Difference in β-diversity was observed between groups
Wang et al., 2022 [26]	United States	361 women with or at high risk of HIV (67% HIV positive) 737 women and men with or at high risk of HIV for lipidomic and metabolomic profiling 112 participants developed carotid artery plaques	CAS	Cross-sectional	16S rRNA sequencing	*Fusobacterium* and *Proteus*	*Odoribacter*	*Fusobacterium* and *Proteus* were positively correlated with a broad set of plasma lipid and metabolite profiles, which grouped into 8 distinct co-expression network modules Two lipid modules, one comprised of lysophosphatidylcholines (LPCs) and lysophosphatidylethanolamines (LPEs), and another enriched in diglyycerides (DGs), were significantly associated with a higher risk of carotid artery plaque formation Phospholipases A1 and A2 were identified as key enzymes in LPC and LPE production No significant difference in α- or β-diversity observed between groups
Wang et al., 2023 [27]	United States	433 women with or at high risk of HIV (65% HIV positive)	Carotid artery plaque	Cross-sectional	Metagenomic shotgun sequencing	*Fusobacterium nucleatum*	*Roseburia hominis*, *Roseburia inulinivorans*, *Johnsonella ignava*, *Odoribacter splanchnicus*, *Clostridium saccharolyticum*	Increased abundance of *F. nucleatum* was associated with carotid plaque and higher levels of CXCL9 (inflammatory marker); Decreased abundance of Roseburia spp. and others inversely associated with CX3CL1; Plasma metabolite imidazole-propionate positively linked with plaque and inflammation No significant difference in α- or β-diversity observed between groups

### β-Diversity

Several studies have reported significant alterations in β-diversity, indicating differences in microbial community composition between patients with ACVD or CAS and controls. Ji et al. (2021) [16], Huang et al. (2023) [18], and Baragetti et al. (2021) [22] consistently demonstrated, despite of no between-group difference in α-diversity, distinct clustering of gut microbiota compositions based on disease status using UniFrac or Bray–Curtis metrics. These results indicate that ACVD is associated with compositional remodeling of the gut microbial environment, even when within-sample diversity is maintained. However, many other studies, such as those by Chen et al. (2021) [15], Jie et al. (2017) [17], and Stø et al. (2022) [23], etc. found no difference in β-diversity between individuals with ACVD/CAS and those without, suggesting that alterations in specific taxa, rather than changes in whole community, may better capture atherosclerotic disease-associated dysbiosis (Table 1).

### Specific Microbial Taxa Associated with Clinical/Subclinical ACVD

Several taxa consistently differ between patients with ACVD/CAS and controls (Table 1). Enrichment of pro-inflammatory and pathogenic taxa, including *Escherichia coli*, *Klebsiella spp.*, *Streptococcus spp.*, and *Ruminococcus gnavus*, were repeatedly observed in ACVD and CAS population. These taxa are known for their roles in inflammation and atherosclerosis promotion owing to their lipopolysaccharide (LPS) [28, 29] In contrast, SCFA-producing and anti-inflammatory taxa such as *Faecalibacterium prausnitzii*, *Roseburia spp.*, *Bacteroides spp.*, and *Eubacterium eligens* were consistently depleted in patients with ACVD or CAS specifically [17, 30, 31]. Reduced abundance of these taxa may impair butyrate synthesis, jeopardizing gut barrier integrity and promoting systemic inflammation. In addition, lower abundance of *Prevotella* species has been consistently observed in individuals with ACVD/CAS across studies. Experimental evidence suggests that certain Bacteroides species, such as *B. vulgatus* and *B. dorei*, may exert protective effects against atherosclerosis by reducing gut-derived LPS production [32]. Although *Prevotella* has been implicated in atherosclerosis through its potential to generate trimethylamine, its overall depletion in patient groups compared with controls suggests that its role may be context-dependent and influenced by host metabolic or dietary factors [33].

Collectively, these findings highlight that atherosclerosis is associated with selective enrichment of pro-inflammatory taxa and depletion of anti-inflammatory taxa. Nonetheless, there was also substantial variability in findings across studies, likely due to differences in geography, diet, lifestyle, and disease stage (e.g., subclinical versus clinical ACVD or stroke).

## Potential Mechanisms of Gut Microbiome Alterations, their Interactions with Other Multi-Omics Layers, and the Development of ACVD

### Gut Microbiome and Metabolomics

Current metabolomics studies demonstrate that gut-derived metabolites are strongly linked to atherosclerosis burden and its underlying biological mechanisms, with evidence from both untargeted metabolomics and targeted analyses of specific microbial metabolites.

In a landmark metabolomics study, Wang et al. (2011) [34] first demonstrated a potential causal link between gut microbiota-derived metabolites and CVD using an unbiased plasma metabolomics approach. By profiling over 2,000 small molecules, they identified that choline, betaine, and TMAO strongly predicted risk of CVD, including myocardial infarction and stroke, independent of traditional risk factors. Experimental validation in mice revealed that dietary supplementation with choline or TMAO markedly enhanced atherosclerotic plaque formation, whereas suppression of gut microbiota using antibiotics abolished this effect.

In a trans-omics study of Chinese adults, Li et al. (2021) [14] integrated stool metagenomics, serum and urine metabolomics, and clinical data, showing that metabolite-based models outperformed microbial taxonomy alone for discriminating CAS. For example, adding urine metabolite ME71, which was lower in CAS patients, improved classification performance to an area under the curve of 0.78, indicating that metabolites may more directly capture plaque-related biological activity than gut microbial composition alone.

A multi-omics study from the MACS/WIHS Combined Cohort (MWCCS) reported positive associations of *Fusobacterium* and *Proteus* with carotid plaque, and an inverse correlation for butyrate-producing *Odoribacter*. Network-based correlation analyses identified eight metabolite-lipid metabolite modules, of which clusters rich in lysophosphatidylcholines (LPCs), lysophosphatidylethanolamines (LPEs), and diglycerides (DGs) were associated with increased risk of carotid plaque development. Functional annotation of microbial genes identified phospholipase A1 and A2 as key bacterial enzymes catalyzing reactions that generate these pro-atherogenic lipid species, providing a mechanistic link bridge between microbial metabolism and host vascular lipid regulation [26].

**Fig. 1:**
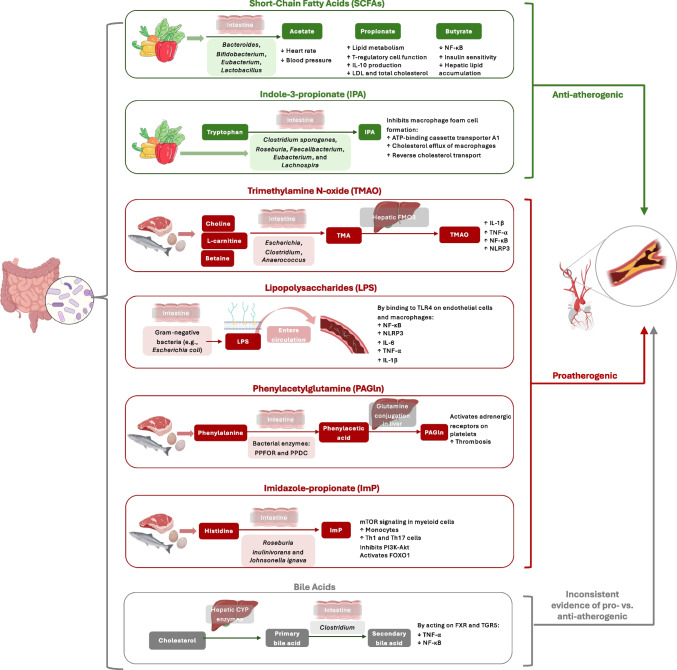
Potential mechanisms for the association between gut microbiota and atherosclerosis. IL: Interleukin; LDL: Low-density lipoprotein (cholesterol); NF-κB: Nuclear factor kappa-light-chain-enhancer of activated B cells; TNF: Tumor necrosis factor; NLRP3: Nucleotide-binding and oligomerization domain (NOD)-like receptors (NLRs) and pyrin domain-containing protein 3; FMO3: Flavin-dependent monooxygenase 3; PPFOR: Phenylpyruvate ferredoxin oxidoreductase; PPDC: Phenylpyruvate decarboxylase; mTOR: Mammalian target of rapamycin; Th: T helper cells; FXR: Farnesoid X receptor; PI3K-Akt: Phosphoinositide 3-kinase (PI3K)/protein kinase B (Akt) pathway; FOXO1: Forkhead box O1

Collectively, metabolomic and metagenomic data reveal that atherosclerosis is influenced by a complex network of gut microbial metabolites that modulate host lipid, amino acid, and inflammatory pathways. Among these, TMAO, SCFAs, bile acids, lipopolysaccharides (LPS), phenylacetylglutamine (PAGln) represent the most well-established mediators of the gut microbiota-atherosclerosis axis [34–39], with more evidence implicating indole-3-propionate (IPA) and imidazole-propionate (ImP) [27, 40]. The following sections and the Fig. 1 discuss these key metabolites in greater mechanistic detail.

#### TMAO

TMAO is one of the most well-studied gut microbiota-derived metabolites consistently shown to be pro-atherogenic. It is generated through a two-step process linking diet, the gut microbiota, and host metabolism [41]. Dietary nutrients rich in trimethylamine (TMA) moieties, such as choline, L-carnitine, and betaine from red meat, eggs, and seafood, are first converted by gut bacteria including *Escherichia*, *Clostridium*, and *Anaerococcus* into TMA [34, 41]. TMA is then oxidized into TMAO by hepatic flavin-dependent monooxygenase 3 (FMO3) [41–43]. Both human and animal studies consistently show that elevated TMAO is positively associated with CVD and carotid intima-media thickness (i.e., an indicator of atherosclerotic burden) [41, 44]. Experimental models have shown that dietary choline or betaine increases macrophage foam cell formation, whereas suppression of gut microbiota markedly reduces this effect [41]. Inhibition of FMO3 by methimazole in mice has been shown to lower TMAO concentrations and significantly reduce carotid plaque area [44]. Mechanistically, TMAO upregulates pro-inflammatory cytokines such as interleukin (IL)-1β, tumor necrosis factor-α (TNF-α), and C-reactive protein via MAPK and nuclear factor (NF)-kB signaling pathways, thereby amplifying vascular inflammation, and it also activates Nucleotide-binding and oligomerization domain (NOD)-like receptors (NLRs) and pyrin domain-containing protein 3 (NLRP3) inflammasome that further impair plaque stability [45].

#### Bile Acid

Bile acids maybe another crucial link between gut microbiota and atherosclerosis, though their role remains complex and requires further investigation In the liver, cholesterol is converted into primary bile acids (cholic acid and chenodeoxycholic acid) through CYP enzyme-mediated reactions [46]. The gut microbiota modulates these processes by regulating CYP7A1, CYP7B1, and CYP27A1 expression. In the intestine, primary bile acids are deconjugated and converted into secondary bile acids (lithocholic acid, deoxycholic acid) by bacterial enzymes such as bile salt hydrolases and hydroxysteroid dehydrogenases, primarily expressed by *Clostridium species* [46–48]. Secondary bile acids act on the nuclear farnesoid X receptor (FXR) and the membrane receptor TGR5, influencing lipid, glucose, and inflammatory pathways [49]. FXR activation in the liver and intestine modulates triglyceride synthesis, very-low-density lipoprotein (VLDL) production, and reverse cholesterol transport, whereas TGR5 signaling can suppress inflammatory cytokine expression, promote cholesterol efflux, and regulate glucose metabolism by increasing incretin secretion [35, 50, 51]. However, the net impact of bile acid-mediated signaling on atherosclerosis remains controversial. Although FXR-deficient mice exhibit worsened plasma lipid profiles and increased atherosclerotic lesion formation, which supports a protective role for FXR, other studies have reported that FXR deletion reduces LDL cholesterol and plaque burden, suggesting that its effects may not be uniformly protective [52, 53]. Moreover, FXR is also expressed in vascular smooth muscle and endothelial cells, where it may directly modulate local inflammatory responses and plaque stability [54]. Overall, while bile acid metabolism has been found to influence FXR and TGR5 signaling, the directional of these effects on atherosclerosis remains unclear.

#### SCFA

SCFAs, including acetate, propionate, and butyrate, are key microbial fermentation products of dietary fiber, produced mainly by *Bacteroides*, *Bifidobacterium*, *Eubacterium,* and *Lactobacillus* [37, 55]. They maintain gut homeostasis and exhibit systemic cardioprotective effects. Acetate can transiently lower heart rate and blood pressure, while propionate improves lipid metabolism and immune regulation by enhancing T-regulatory cell function and IL-10 production, thereby lowering LDL and total cholesterol [56–58]. Butyrate suppresses NF-kB activity, enhances insulin sensitivity, reduces hepatic lipid accumulation, and prevents diet-induced atherosclerosis [59–61]. SCFAs also support colonocyte β-oxidation and contribute to the low pH environment of the gut lumen, maintaining anaerobic conditions that suppress pathogen growth and preserve beneficial anaerobes [62]. Collectively, these effects highlight the anti-inflammatory and metabolic benefits of SCFAs in vascular health.

#### LPS

LPS, an endotoxin derived from the outer membrane of Gram-negative bacteria, represents a key pro-inflammatory mediator linking dysbiosis to vascular injury [38]. Gut barrier disruption allows translocation of LPS into circulation, inducing chronic low-grade inflammation [63]. Once in the bloodstream, LPS binds to toll-like receptor 4 (TLR4) on endothelial cells and macrophages, activating NF-kB and NLRP3 inflammasome pathways and promoting secretion of IL-6, TNF-α, and IL-1β [63]. These cytokines enhance endothelial dysfunction, monocyte adhesion, and plaque formation. Both animal studies and human cohort studies have shown that circulating LPS levels are positively associated with CAS severity [64–67].

#### PAGln

PAGln, a metabolite of phenylalanine catabolism, has recently emerged as another microbial driver of atherosclerosis [39]. In the gut, phenylalanine is converted by bacterial enzymes (i.e., phenylpyruvate decarboxylase [PPDC] and phenylpyruvate ferredoxin oxidoreductase [PPFOR]) into phenylacetic acid, which is then conjugated with glutamine in the liver to form PAGln [39]. Metagenomic studies demonstrated that genes encoding PPDC and PPFOR are enriched in the gut microbiota of patients with atherosclerotic disease [68]. PAGln activates adrenergic receptors on platelets, enhancing thrombosis and vascular inflammation, while also promoting oxidative stress and endothelial dysfunction [39, 69].

#### IPA

IPA is a microbial-derived metabolite from tryptophan [70]. Emerging evidence has demonstrated its anti-atherogenic effects, and circulating levels of IPA were positively associated with high-fiber diets, likely through enrichment of IPA-producing gut microbiota [40, 70, 71]. The known IPA producers include *Clostridium sporogenes*, while other taxa such as *Roseburia*, *Faecalibacterium*, *Eubacterium*, and *Lachnospira* have shown positive associations with host circulating IPA levels in human studies [40, 72]. In the Women’s Interagency HIV Study (WIHS) cohort, plasma IPA was inversely associated with carotid plaque, independent of HIV serostatus [40]. In murine models, IPA supplementation reduced atherosclerotic plaque burden, lipid accumulation within plaques, and plasma total cholesterol and triglyceride levels [73]. Mechanistically, IPA inhibits macrophage foam cell formation by promoting reverse cholesterol transport, enhancing cholesterol efflux to apolipoprotein A-I (ApoA-I) through upregulation of ATP-binding cassette transporter A1 (ABCA1), without altering ABCG1 or SR-BI expression [73].

#### ImP

ImP is a microbial-derived metabolite from histidine [74]. In the WIHS cohort, ImP was elevated in women with carotid plaque and inversely correlated with potentially beneficial bacterial species such as *Roseburia inulinivorans* and *Johnsonella ignava* [27]. Consistent findings were recently reported by another study that investigated potential mechanisms of ImP contributing to atherosclerosis [75]. ImP has been shown to promote atherosclerosis through activation of an imidazole-1 receptor (I1R)-mTOR signaling path in myeloid cells [75]. ImP supplementation in mouse models has been shown to increase aortic plaque burden without altering circulating cholesterol or glucose levels, and it induced expansion of pro-inflammatory immune cells such as Ly6C^hi^ monocytes and T helper (Th) 1 and Th17 cells, as well as activated inflammatory and lipid metabolic pathways in macrophages, fibroblasts, and endothelial cells [75]. Additionally, recent evidence has shown that ImP impairs endothelial repair via PI3K-AKT inhibition and FOXO1 activation [76].

### Microbial Functional Components, Proteomics, and Host Genetics

Proteomics studies also support the link between gut microbiota and the biological mechanisms underlying atherogenesis. In the WIHS cohort, shotgun metagenomic and plasma proteomic profiling showed that microbial composition was closely connected to systemic inflammation and carotid plaque burden [27]. Pro-atherogenic taxa such as *Fusobacterium nucleatum* were positively associated with circulating inflammatory biomarkers (e.g., CXCLL9), while anti-atherogenic taxa, including SCFA-producing bacteria (e.g., *Roseburia* and *Faecalibacterium*), were inversely associated with inflammatory proteins CX3CL1 and LIF-R. Notably, adding plasma proteomic markers to statistical models attenuated associations between microbial taxa and carotid artery plaque, suggesting inflammation as a major mediator linking gut microbial dysbiosis to CAS [27].

Functional metagenomic analysis further revealed that the microbial gene repertoire is altered in CAS. In one of the first shotgun metagenomic analyses of patients with symptomatic CAS, Karlsson et al. demonstrated enrichment of peptidoglycan biosynthesis genes involved in proinflammatory bacterial functions, as well as depletion of phytoene dehydrogenase, a gene involved in β-carotene and carotenoid metabolism [19]. Patients also showed lower serum β-carotene levels, linking the microbial gene changes to host antioxidant status. These findings suggest that functional shifts within the gut microbiome toward proinflammation, despite an enrichment in butyrate-producing bacteria such as *Clostridium spp*. [19].

Beyond inflammation, large population-based proteomic analyses have demonstrated that gut microbiota is linked to host neuroendocrine proteins. In the LifeLines-DEEP cohort of over 1,000 Dutch adults, microbial diversity was associated with fecal Chromogranin A (CgA), which is a neuroendocrine peptide released by enteroendocrine cells that reflects mucosal immune activation and epithelial integrity [76, 77]. Individuals with lower CgA exhibited a more diverse and metabolically favorable microbiome [77]. Complementary evidence from the LifeLines-DEEP systems-genomic study further revealed that host genetics and gut microbiota had additive effects on circulating proteins related to cardiovascular disease, collectively explaining up to 77% of inter-individual variation. Notably, microbial features were most strongly linked to proteins for lipid metabolism and fibrinolysis, such as PON3 that links to HDL level and suppresses LDL oxidation in the blood and showed the strongest association to α- and β-diversity, and plasminogen activator inhibitor (PAI)-1 [78]. These findings underscore the integrated host-microbe regulation of vascular and metabolic homeostasis.

Consistently, an integrated multi-omics study of CAS combined 16S rRNA sequencing, untargeted metabolomics, and transcriptomics to identify a coordinated microbiota-metabolite-gene network [16]. Patients with CAS displayed an upregulation of *FABP4* gene expression that encodes fatty acid binding protein 4, which is involved in macrophage activation and lipid metabolism in atherosclerosis [79]. Notably, *FABP4* expression was also correlated with *Acidaminococcus* and PAGln, suggesting a potential gut microbial influence on lipid metabolism and inflammation via the interplay between microbiota, metabolites, and host genetics [16]. Together, these findings show how genetic, microbial, proteomics, and transcriptomics converge to implicate CAS risk and progression.

## Other Modifying Factors

The relationship between the gut microbiome and ACVD is modulated by a range of other factors, such as sex, age, and diet. Females and males differ not only in cardiovascular physiology but also in their gut microbial composition and metabolite production [79, 80]. It has been shown that females have higher levels of *Bifidobacterium*, *Ruminococcus*, and *Akkermansia*, while males have higher abundance of *Prevotella*, *Megamonas*, and *Fusobacterium* [81]. Emerging evidence indicates that sex hormones actively shape the gut microbiome, because no sex difference was observed in early childhood or until puberty, likely due that gonadal hormones have not been initiated [82]. For example, it has been shown that estrogen promotes the growth of *Akkermansia*, which produces SCFAs, and *Prevotella* was found to be positively associated with testosterone levels [83]. Sex has also been shown to differentially impact the TMAO synthesis, where both the expression of FMO3, which is the key microbial enzyme that converts TMA to TMAO, is higher in females and males in both human and mouse studies, and the plasma level of TMAO in female has also been shown higher than males in mouse studies [8]. These sex-differences in gut microbial composition and metabolite production may contribute to variations in atherosclerosis susceptibility between males and females. Male-enriched taxa such as *Fusobacterium* have been linked to proinflammatory pathways and altered lipid metabolism, whereas female-enriched SCFA-producing bacteria may confer partial protection through anti-inflammatory effects [84]. Moreover, given that TMAO is a well-known player in atherosclerosis pathophysiology, sex-differences in TMAO synthesis and metabolism could further explain the observed difference in atherosclerosis risk.

Aging comes with a gradual decline in gut microbial diversity and a shift toward taxa associated with inflammation and metabolic dysfunction. As people age, gut microbiota diversity tends to decrease [85]. However, exceptionally long-lived individuals with age above 100 years old often retain high microbial diversity and abundance of health-associated taxa including *Christensenellaceae*, *Lachnospiraceae*, *Ruminococcaceae*, and *Akkermansia*, which primarily produce SCFAs [85]. Importantly, dietary intervention can mitigate age-related microbial deterioration. In the large multicenter NU-AGE trial, a one-year Mediterranean diet intervention in older adults across five European countries led to increased abundance of beneficial SCFA-producing bacteria and reduced inflammatory markers like C-reactive protein and IL-17 [86]. Participants with greater adherence to the Mediterranean diet showed improved frailty scores and cognitive function, suggesting that targeted dietary modulation of the gut microbiota may promote healthier aging and reduce inflammation-linked vascular risk [86].

## Research Gaps and Future Directions

Although substantial evidence now links gut microbial dysbiosis and microbially derived metabolites with atherosclerosis, critical knowledge gaps remain before these findings can be translated into clinical interventions. Current studies are largely cross-sectional, limiting causal inference. Future research should prioritize longitudinal and interventional study designs integrating microbial metagenomics and metabolomics together with host multi-omics and clinical characteristics to delineate causal relationships between microbiome alterations and plaque development, particularly in CAS, which directly contributes to ischemic stroke risk.

Probiotics may be a potential approach to modulate atherosclerosis-related dysbiosis, but the responses to probiotics or potential metabolite drug candidates depend heavily on an individual’s baseline microbiome, metabolic profile, inflammatory state, dietary intake, and medication use. Future studies should determine whether precision interventions, such as stratifying individuals by their gut microbiota types, diversities, and metabolite profiles, are feasible and clinically meaningful. Given the heterogeneity in responses to probiotics, integrating host- and microbiome-specific information into intervention design might be informative. Combining dietary modulation with targeted microbial or metabolic interventions may offer a precision-medicine approach to atherosclerosis prevention [62]. Also, because probiotic effects are strain-specific, future work should determine and validate which strains have metabolic or immunomodulatory effects to atherosclerosis.

Fecal microbiota transplantation (FMT) has been shown to restore microbial diversity in conditions like *Clostridioides difficile* infection, but its application to atherosclerosis remains underexplored. Evidence from mouse studies shows that transferring the gut microbiota from healthy wild-type donors into atherosclerosis-prone mice can attenuate carotid plaque formation [87]. However, in cardiometabolic diseases, FMT has generally produced modest, transient, and highly variable effects [62]. Future research should determine whether more targeted or optimized FMT approaches can produce durable and reproducible benefits specifically for atherosclerosis.

Emerging metabolites, such as ImP, may be a novel therapeutic target. Elevated ImP has been consistently observed in individuals with insulin resistance and type 2 diabetes [74]. Recent mechanistic studies demonstrate that ImP promotes atherosclerosis through activation of imidazole-1 receptor in myeloid cells, suggesting a new metabolic-immune axis in vascular damage [75]. Future studies should investigate whether targeting ImP signaling can reduce cardiometabolic and atherosclerotic risk, particularly among individuals with metabolic disorders or those treated with GLP-1 receptor agonists. This is especially relevant because the prevalence of GLP-1 utilization has been increasing, and ImP has been shown to impair the glucose-lowering efficacy of metformin, a drug, like GLP-1 RAs, improves insulin sensitivity [86, 87].

Hyperlipidemia represents another promising area for microbiome-targeted therapy. Clinical and preclinical studies show that probiotic and symbiotic supplementation, such as those with *Lactobacillus* and *Bifidobacterium*, can improve lipid profiles [62]. However, these studies are often limited by small sample sizes, short intervention durations, and inconsistent formulations, and therefore larger randomized controlled trials are needed to establish efficacy, optimizing dosing, and assess long-term effects and safety.

Beyond probiotics, pharmacologic inhibition of microbial enzymes may also be a translational route. For instance, non-lethal inhibition of TMA lyase, which is the enzyme responsible for the first step in TMAO production from dietary choline, has been shown in animal models to prevent macrophage foam cell formation and reduce plaque burden without altering cholesterol levels [88]. However, such promising results still need to be validated in human trials. Similarly, bile acid-responsive receptors like FXR have emerged as a potential therapeutic target, but contradictory evidence has been shown for its role on atherosclerosis, and thus more research is needed [89].

Microbiota-derived metabolites could also serve as novel biomarkers for early detection of CAS. As shown in recent multi-omics studies, urinary metabolites can outperform microbial taxonomy alone in distinguishing individuals with carotid plaque, highlighting the diagnostic potential of metabolomic signatures [14]. Standardizing and pipelining the analytical methods for metabolite profiling and integrating these data with host genetics and proteomics may accelerate translating these findings into clinical applications for risk stratification.

## Conclusion

Accumulating evidence indicates that the gut microbiota can act as both a modulator and a biomarker of ACVD. Microbial dysbiosis may contribute to ACVD pathogenesis through multiple mechanisms, most notably via microbiota-derived metabolites that influence endothelial function, systemic inflammation, and lipid metabolism. Alterations in the abundance of specific microbial taxa, characterized by enrichment of pro-inflammatory species and depletion of anti-inflammatory, SCFA-producing bacteria, further underscore the microbiota’s mechanistic involvement in vascular injury.

Despite these advances, considerable heterogeneity persists across studies. Future longitudinal and interventional research integrating metagenomics and host multi-omics is essential to clarify causal relationships and identify clinically actionable targets. With further evidence, leveraging microbiome-based biomarkers and interventions, potentially through personalized nutrition, probiotics, or modulation of specific microbial functional enzymes, may enable precision prevention and treatment strategies for ACVD and broader cardiovascular diseases.

## Key References

- Ji L, Chen S, Gu G, Zhou J, Wang W, Ren J, et al. Exploration of Crucial Mediators for Carotid Atherosclerosis Pathogenesis Through Integration of Microbiome, Metabolome, and Transcriptome. Front Physiol. 2021;12:645212. 10.3389/fphys.2021.645212
  - ○ This study integrated microbiome, metabolome, and transcriptome analyses to provide mechanistic insight into the link between gut microbiota and carotid atherosclerosis.
- Szabo H, Hernyes A, Piroska M, Ligeti B, Fussy P, Zoldi L, et al. Association between Gut Microbial Diversity and Carotid Intima-Media Thickness. Medicina (Kaunas). 2021;57:195. 10.3390/medicina57030195
  - ○ This study used monozygotic twins to control for genetic factors and demonstrated gut microbial alterations associated with increased carotid intima-media thickness, highlighting a potential microbial contribution to subclinical atherosclerosis.
- Zhu S, Xu K, Jiang Y, Zhu C, Suo C, Cui M, et al. The gut microbiome in subclinical atherosclerosis: a population-based multiphenotype analysis. Rheumatology (Oxford). 2022;61:258–69. 10.1093/rheumatology/keab309
  - ○ This large population-based study characterized gut microbiome features associated with subclinical atherosclerosis across multiple phenotypes, demonstrating that microbial composition and diversity were linked to carotid plaque and vascular risk profiles in asymptomatic individuals.
- Wang Z, Peters BA, Usyk M, Xing J, Hanna DB, Wang T, et al. Gut Microbiota, Plasma Metabolomic Profiles, and Carotid Artery Atherosclerosis in HIV Infection. Arteriosclerosis, Thrombosis, and Vascular Biology. Lippincott Williams & WilkinsHagerstown, MD; 2022; 10.1161/ATVBAHA.121.317276
  - ○ This study integrated gut microbiome and plasma metabolomics data and revealed that specific gut microbes were associated with altered lipid metabolism and carotid artery atherosclerosis, highlighting a potential microbe-lipid-atherosclerosis axis.
- Wang Z, Peters BA, Bryant M, Hanna DB, Schwartz T, Wang T, et al. Gut microbiota, circulating inflammatory markers and metabolites, and carotid artery atherosclerosis in HIV infection. Microbiome. 2023;11:119. 10.1186/s40168-023-01566-2
  - ○ This paper integrated microbiome, metabolome, and proteomic inflammation marker data, identified specific gut bacterial species, as well as the microbial metabolite imidazole propionate (ImP), that were associated with carotid artery atherosclerosis, potentially through mechanisms involving host immune activation and inflammation.
- Luo K, Wang Z, Peters BA, Hanna DB, Wang T, Sollecito CC, et al. Tryptophan metabolism, gut microbiota, and carotid artery plaque in women with and without HIV infection. AIDS. 2024;38:223–33. 10.1097/QAD.0000000000003596
  - ○ This study provided evidence showing that higher levels of indole-3-propionate (IPA) were inversely associated with carotid artery plaque and also reported the gut microbial species related to IPA production.
- Mastrangelo A, Robles-Vera I, Mañanes D, Galán M, Femenía-Muiña M, Redondo-Urzainqui A, et al. Imidazole propionate is a driver and therapeutic target in atherosclerosis. Nature. Nature Publishing Group; 2025;645:254–61. 10.1038/s41586-025-09263-w
  - ○ This study provided the most recent evidence showing that circulating imidazole propionate levels are associated with coronary artery diseases, suggesting its potential as both a biomarker and a therapeutic effect.

## Data Availability

No datasets were generated or analysed during the current study.

## References

[1] Global, Regional, and National Burden of Cardiovascular Diseases and Risk Factors in 204 Countries and Territories. 1990–2023. JACC [Internet]. 2025[cited 2025 Oct 26]. 10.1016/j.jacc.2025.08.015. 10.1016/j.jacc.2025.08.015 40990886

[2] Bir SC, Kelley RE. Carotid atherosclerotic disease: a systematic review of pathogenesis and management. Brain Circ. 2022;8:127–36. 10.4103/bc.bc_36_22. 36267431 10.4103/bc.bc_36_22 PMC9578307

[3] Johri AM, Nambi V, Naqvi TZ, Feinstein SB, Kim ESH, Park MM, et al. Recommendations for the assessment of carotid arterial plaque by ultrasound for the characterization of atherosclerosis and evaluation of cardiovascular risk: from the american society of echocardiography. J Amer Soc Echocardiogr Elsevier. 2020;33:917–33. 10.1016/j.echo.2020.04.021. 10.1016/j.echo.2020.04.021 32600741

[4] Song P, Fang Z, Wang H, Cai Y, Rahimi K, Zhu Y, et al. Global and regional prevalence, burden, and risk factors for carotid atherosclerosis: a systematic review, meta-analysis, and modelling study. Lancet Global Health Elsevier. 2020;8:e721–9. 10.1016/S2214-109X(20)30117-0. 10.1016/S2214-109X(20)30117-0 32353319

[5] Mahmood SS, Levy D, Vasan RS, Wang TJ. The framingham heart study and the epidemiology of cardiovascular diseases: a historical perspective. Lancet. 2014;383:999–1008. 10.1016/S0140-6736(13)61752-3. 24084292 10.1016/S0140-6736(13)61752-3 PMC4159698

[6] Atherosclerosis - Causes and Risk Factors | NHLBI, NIH [Internet]. 2024 [cited 2025 Oct 19]. https://www.nhlbi.nih.gov/health/atherosclerosis/causes. Accessed 19 Oct 2025.

[7] Kuo F, Gardener H, Dong C, Cabral D, Della-Morte D, Blanton SH, et al. Traditional cardiovascular risk factors explain the minority of the variability in carotid plaque. Stroke. 2012;43:1755–60. 10.1161/STROKEAHA.112.651059. 22550054 10.1161/STROKEAHA.112.651059 PMC3383876

[8] Jonsson AL, Bäckhed F. Role of gut microbiota in atherosclerosis. Nat Rev Cardiol. 2017;14:79–87. 10.1038/nrcardio.2016.183. 27905479 10.1038/nrcardio.2016.183

[9] Roth W, Lo E, De Leon O, Suriya S, Fakhri F, Brorson JR, et al. Understanding the relationship between cerebrovascular disease and the gut microbiome. Stroke Vasc Interv Neurol. 2025;5:e001272. 10.1161/SVIN.124.001272. 10.1161/SVIN.124.001272 PMC11864580 41608402

[10] Joh JH, Cho S. Cardiovascular risk of carotid atherosclerosis: global consensus beyond societal guidelines. Lancet Glob Health. 2020;8:e625–6. 10.1016/S2214-109X(20)30132-7. 32353304 10.1016/S2214-109X(20)30132-7

[11] Laksono S, Kusharsamita H. Unravelling the role of carotid atherosclerosis in predicting cardiovascular disease risk: a review. ARYA Atherosclerosis Journal. 2024;20:52–9. 10.48305/arya.2024.41271.2862. 10.48305/arya.2024.41271.2862 PMC11663449 39717161

[12] Qin J, Li R, Raes J, Arumugam M, Burgdorf KS, Manichanh C, et al. A human gut microbial gene catalogue established by metagenomic sequencing. Nature. 2010;464:59–65. 10.1038/nature08821. 20203603 10.1038/nature08821 PMC3779803

[13] Haak BW, Westendorp WF, van Engelen TSR, Brands X, Brouwer MC, Vermeij J-D, et al. Disruptions of anaerobic gut bacteria are associated with stroke and post-stroke infection: a prospective case-control study. Transl Stroke Res. 2021;12:581–92. 10.1007/s12975-020-00863-4. 33052545 10.1007/s12975-020-00863-4 PMC8213601

[14] Li R-J, Jie Z-Y, Feng Q, Fang R-L, Li F, Gao Y, et al. Network of interactions between gut microbiome, host biomarkers, and urine metabolome in carotid atherosclerosis. Front Cell Infect Microbiol. 2021. 10.3389/fcimb.2021.708088. 34692558 10.3389/fcimb.2021.708088 PMC8529068

[15] Chen J, Qin Q, Yan S, Yang Y, Yan H, Li T, et al. Gut microbiome alterations in patients with carotid atherosclerosis. Front Cardiovasc Med. 2021;8:739093. 10.3389/fcvm.2021.739093. 34869642 10.3389/fcvm.2021.739093 PMC8639581

[16] Ji L, Chen S, Gu G, Zhou J, Wang W, Ren J, et al. Exploration of crucial mediators for carotid atherosclerosis pathogenesis through integration of microbiome, metabolome, and transcriptome. Front Physiol. 2021;12:645212. 10.3389/fphys.2021.645212. 34108883 10.3389/fphys.2021.645212 PMC8181762

[17] Jie Z, Xia H, Zhong S-L, Feng Q, Li S, Liang S, et al. The gut microbiome in atherosclerotic cardiovascular disease. Nat Commun. 2017;8:845. 10.1038/s41467-017-00900-1. 29018189 10.1038/s41467-017-00900-1 PMC5635030

[18] Huang H, Kuang Z, Mo R, Meng M, Cai Y, Ni X, et al. The preliminary evidence on the association of the gut microbiota with stroke risk stratification in South Chinese population. Front Cell Infect Microbiol. 2023. 10.3389/fcimb.2023.1227450. 38222855 10.3389/fcimb.2023.1227450 PMC10785002

[19] Karlsson FH, Fåk F, Nookaew I, Tremaroli V, Fagerberg B, Petranovic D, et al. Symptomatic atherosclerosis is associated with an altered gut metagenome. Nat Commun. 2012;3:1245. 10.1038/ncomms2266. 23212374 10.1038/ncomms2266 PMC3538954

[20] Szabo H, Hernyes A, Piroska M, Ligeti B, Fussy P, Zoldi L, et al. Association between gut microbial diversity and carotid intima-media thickness. Medicina. 2021;57:195. 10.3390/medicina57030195. 33668894 10.3390/medicina57030195 PMC7996485

[21] Zhu S, Xu K, Jiang Y, Zhu C, Suo C, Cui M, et al. The gut microbiome in subclinical atherosclerosis: a population-based multiphenotype analysis. Rheumatology. 2022;61:258–69. 10.1093/rheumatology/keab309. 10.1093/rheumatology/keab309 33769467

[22] Baragetti A, Severgnini M, Olmastroni E, Dioguardi CC, Mattavelli E, Angius A, et al. Gut microbiota functional dysbiosis relates to individual diet in subclinical carotid atherosclerosis. Nutrients. 2021;13:304. 10.3390/nu13020304. 33494335 10.3390/nu13020304 PMC7911134

[23] Stø K, Valeur J, Ueland T, Malmstrøm GH, Bjerkeli V, Trøseid M, et al. Fecal level of butyric acid, a microbiome-derived metabolite, is increased in patients with severe carotid atherosclerosis. Sci Rep. 2022;12:22378. 10.1038/s41598-022-26759-x. 36572703 10.1038/s41598-022-26759-x PMC9792531

[24] Li H, Zhang X, Pan D, Liu Y, Yan X, Tang Y, et al. Dysbiosis characteristics of gut microbiota in cerebral infarction patients. Transl Neurosci. 2020;11:124–33. 10.1515/tnsci-2020-0117. 33312718 10.1515/tnsci-2020-0117 PMC7706127

[25] Tan C, Wu Q, Wang H, Gao X, Xu R, Cui Z, et al. Dysbiosis of gut microbiota and short-chain fatty acids in acute ischemic stroke and the subsequent risk for poor functional outcomes. J Parenter Enteral Nutr. 2021;45:518–29. 10.1002/jpen.1861. 10.1002/jpen.1861 PMC8048557 32473086

[26] Wang Z, Peters BA, Usyk M, Xing J, Hanna DB, Wang T, et al. Gut microbiota, plasma metabolomic profiles, and carotid artery atherosclerosis in HIV infection. Arterioscler Thromb Vasc Biol. 2022. 10.1161/ATVBAHA.121.317276. 35678187 10.1161/ATVBAHA.121.317276 PMC9339474

[27] Wang Z, Peters BA, Bryant M, Hanna DB, Schwartz T, Wang T, et al. Gut microbiota, circulating inflammatory markers and metabolites, and carotid artery atherosclerosis in HIV infection. Microbiome. 2023;11:119. 10.1186/s40168-023-01566-2. 37237391 10.1186/s40168-023-01566-2 PMC10224225

[28] Carnevale R, Nocella C, Petrozza V, Cammisotto V, Pacini L, Sorrentino V, et al. Localization of lipopolysaccharide from *Escherichia coli* into human atherosclerotic plaque. Sci Rep. 2018;8:3598. 10.1038/s41598-018-22076-4. 29483584 10.1038/s41598-018-22076-4 PMC5826929

[29] Hashizume-Takizawa T, Yamaguchi Y, Kobayashi R, Shinozaki-Kuwahara N, Saito M, Kurita-Ochiai T. Oral challenge with *Streptococcus sanguinis* induces aortic inflammation and accelerates atherosclerosis in spontaneously hyperlipidemic mice. Biochem Biophys Res Commun. 2019;520:507–13. 10.1016/j.bbrc.2019.10.057. 31610917 10.1016/j.bbrc.2019.10.057

[30] Sokol H, Pigneur B, Watterlot L, Lakhdari O, Bermúdez-Humarán LG, Gratadoux J-J, et al. *Faecalibacterium prausnitzii* is an anti-inflammatory commensal bacterium identified by gut microbiota analysis of Crohn disease patients. Proc Natl Acad Sci U S A. 2008;105:16731–6. 10.1073/pnas.0804812105. 18936492 10.1073/pnas.0804812105 PMC2575488

[31] Nie K, Ma K, Luo W, Shen Z, Yang Z, Xiao M, et al. *Roseburia intestinalis*: a beneficial gut organism from the discoveries in genus and species. Front Cell Infect Microbiol. 2021;11:757718. 10.3389/fcimb.2021.757718. 34881193 10.3389/fcimb.2021.757718 PMC8647967

[32] Yoshida N, Emoto T, Yamashita T, Watanabe H, Hayashi T, Tabata T, et al. *Bacteroides vulgatus* and *Bacteroides dorei* reduce gut microbial lipopolysaccharide production and inhibit atherosclerosis. Circulation. 2018;138:2486–98. 10.1161/CIRCULATIONAHA.118.033714. 30571343 10.1161/CIRCULATIONAHA.118.033714

[33] Lau K, Srivatsav V, Rizwan A, Nashed A, Liu R, Shen R, et al. Bridging the gap between gut microbial dysbiosis and cardiovascular diseases. Nutr Multidis Dig Pub Inst. 2017;9:859. 10.3390/nu9080859. 10.3390/nu9080859 PMC5579652 28796176

[34] Wang Z, Klipfell E, Bennett BJ, Koeth R, Levison BS, Dugar B, et al. Gut flora metabolism of phosphatidylcholine promotes cardiovascular disease. Nature. 2011;472:57–63. 10.1038/nature09922. 21475195 10.1038/nature09922 PMC3086762

[35] Salazar J, Morillo V, Suárez MK, Castro A, Ramírez P, Rojas M, et al. Role of gut microbiome in atherosclerosis: molecular and therapeutic aspects. Curr Cardiol Rev. 2023;19:E020223213408. 10.2174/1573403X19666230202164524. 36733248 10.2174/1573403X19666230202164524 PMC10494273

[36] Zhu W, Gregory JC, Org E, Buffa JA, Gupta N, Wang Z, et al. Gut microbial metabolite TMAO enhances platelet hyperreactivity and thrombosis risk. Cell. 2016;165:111–24. 10.1016/j.cell.2016.02.011. 26972052 10.1016/j.cell.2016.02.011 PMC4862743

[37] Ohira H, Tsutsui W, Fujioka Y. Are short chain fatty acids in gut microbiota defensive players for inflammation and atherosclerosis? J Atheroscler Thromb. 2017;24:660–72. 10.5551/jat.RV17006. 28552897 10.5551/jat.RV17006 PMC5517538

[38] Page MJ, Kell DB, Pretorius E. The role of lipopolysaccharide-induced cell signalling in chronic inflammation. Chronic Stress. 2022;6:24705470221076390. 10.1177/24705470221076390. 35155966 10.1177/24705470221076390 PMC8829728

[39] Chen W, Li M-L, Zeng G, Xu X-Y, Yin S-H, Xu C, et al. Gut microbiota-derived metabolite phenylacetylglutamine in cardiovascular and metabolic diseases. Pharmacol Res. 2025;217:107794. 10.1016/j.phrs.2025.107794. 40409519 10.1016/j.phrs.2025.107794

[40] Luo K, Wang Z, Peters BA, Hanna DB, Wang T, Sollecito CC, et al. Tryptophan metabolism, gut microbiota, and carotid artery plaque in women with and without HIV infection. AIDS. 2024;38:223–33. 10.1097/QAD.0000000000003596. 37199567 10.1097/QAD.0000000000003596 PMC10640661

[41] Caradonna E, Abate F, Schiano E, Paparella F, Ferrara F, Vanoli E, et al. Trimethylamine-N-oxide (TMAO) as a rising-star metabolite: implications for human health. Metabolites. 2025;15:220. 10.3390/metabo15040220. 40278349 10.3390/metabo15040220 PMC12029716

[42] Fennema D, Phillips IR, Shephard EA. Trimethylamine and trimethylamine *N*-oxide, a flavin-containing monooxygenase 3 (FMO3)-mediated host-microbiome metabolic axis implicated in health and disease. Drug Metab Dispos. 2016;44:1839–50. 10.1124/dmd.116.070615. 27190056 10.1124/dmd.116.070615 PMC5074467

[43] Al Samarraie A, Pichette M, Rousseau G. Role of the gut microbiome in the development of atherosclerotic cardiovascular disease. Int J Mol Sci. 2023;24:5420. 10.3390/ijms24065420. 36982492 10.3390/ijms24065420 PMC10051145

[44] Shi W, Huang Y, Yang Z, Zhu L, Yu B. Reduction of TMAO level enhances the stability of carotid atherosclerotic plaque through promoting macrophage M2 polarization and efferocytosis. Biosci Rep. 2021;41:BSR20204250. 10.1042/BSR20204250. 33969376 10.1042/BSR20204250 PMC8176787

[45] Zheng S, Liu Z, Liu H, Lim JY, Li DWH, Zhang S, et al. Research development on gut microbiota and vulnerable atherosclerotic plaque. Heliyon. 2024;10:e25186. 10.1016/j.heliyon.2024.e25186. 38384514 10.1016/j.heliyon.2024.e25186 PMC10878880

[46] Collins SL, Stine JG, Bisanz JE, Okafor CD, Patterson AD. Bile acids and the gut microbiota: metabolic interactions and impacts on disease. Nat Rev Microbiol. 2023;21:236–47. 10.1038/s41579-022-00805-x. 36253479 10.1038/s41579-022-00805-x PMC12536349

[47] Wahlström A, Sayin SI, Marschall H-U, Bäckhed F. Intestinal crosstalk between bile acids and microbiota and its impact on host metabolism. Cell Metab. 2016;24:41–50. 10.1016/j.cmet.2016.05.005. 27320064 10.1016/j.cmet.2016.05.005

[48] Yntema T, Koonen DPY, Kuipers F. Emerging roles of gut microbial modulation of bile acid composition in the etiology of cardiovascular diseases. Nutrients. 2023;15:1850. 10.3390/nu15081850. 37111068 10.3390/nu15081850 PMC10141989

[49] Miyazaki-Anzai S, Masuda M, Kohno S, Levi M, Shiozaki Y, Keenan AL, et al. Simultaneous inhibition of FXR and TGR5 exacerbates atherosclerotic formation. J Lipid Res. 2018;59:1709–13. 10.1194/jlr.M087239. 29976576 10.1194/jlr.M087239 PMC6121929

[50] Pols TWH, Nomura M, Harach T, Lo Sasso G, Oosterveer MH, Thomas C, et al. TGR5 activation inhibits atherosclerosis by reducing macrophage inflammation and lipid loading. Cell Metab. 2011;14:747–57. 10.1016/j.cmet.2011.11.006. 22152303 10.1016/j.cmet.2011.11.006 PMC3627293

[51] Hageman J, Herrema H, Groen AK, Kuipers F. A role of the bile salt receptor FXR in atherosclerosis. Arterioscler Thromb Vasc Biol. 2010;30:1519–28. 10.1161/ATVBAHA.109.197897. 20631352 10.1161/ATVBAHA.109.197897

[52] Hanniman EA, Lambert G, McCarthy TC, Sinal CJ. Loss of functional farnesoid X receptor increases atherosclerotic lesions in apolipoprotein E-deficient mice. J Lipid Res. 2005;46:2595–604. 10.1194/jlr.M500390-JLR200. 16186601 10.1194/jlr.M500390-JLR200

[53] Zhang Y, Wang X, Vales C, Lee FY, Lee H, Lusis AJ, et al. FXR deficiency causes reduced atherosclerosis in Ldlr-/- mice. Arterioscler Thromb Vasc Biol. 2006;26:2316–21. 10.1161/01.ATV.0000235697.35431.05. 16825595 10.1161/01.ATV.0000235697.35431.05

[54] Zhang Q, He F, Kuruba R, Gao X, Wilson A, Li J, et al. FXR-mediated regulation of angiotensin type 2 receptor expression in vascular smooth muscle cells. Cardiovasc Res. 2008;77:560–9. 10.1093/cvr/cvm068. 18006431 10.1093/cvr/cvm068

[55] Shen X, Li L, Sun Z, Zang G, Zhang L, Shao C, et al. Gut microbiota and atherosclerosis—focusing on the plaque stability. Front Cardiovasc Med. 2021;8:668532. 10.3389/fcvm.2021.668532. 34414217 10.3389/fcvm.2021.668532 PMC8368126

[56] Poll BG, Xu J, Jun S, Sanchez J, Zaidman NA, He X, et al. Acetate, a short-chain fatty acid, acutely lowers heart rate and cardiac contractility along with blood pressure. J Pharmacol Exp Ther. 2021;377:39–50. 10.1124/jpet.120.000187. 33414131 10.1124/jpet.120.000187 PMC7985618

[57] Haghikia A, Zimmermann F, Schumann P, Jasina A, Roessler J, Schmidt D, et al. Propionate attenuates atherosclerosis by immune-dependent regulation of intestinal cholesterol metabolism. Eur Heart J. 2021;43:518–33. 10.1093/eurheartj/ehab644. 10.1093/eurheartj/ehab644 PMC9097250 34597388

[58] Bartolomaeus H, Balogh A, Yakoub M, Homann S, Markó L, Höges S, et al. Short-chain fatty acid propionate protects from hypertensive cardiovascular damage. Circulation. 2019;139:1407–21. 10.1161/CIRCULATIONAHA.118.036652. 30586752 10.1161/CIRCULATIONAHA.118.036652 PMC6416008

[59] Dicks LMT. Butyrate produced by gut microbiota regulates atherosclerosis: a narrative review of the latest findings. Int J Mol Sci. 2025;26:6744. 10.3390/ijms26146744. 40724991 10.3390/ijms26146744 PMC12295145

[60] Raso GM, Simeoli R, Russo R, Iacono A, Santoro A, Paciello O, et al. Effects of sodium butyrate and its synthetic amide derivative on liver inflammation and glucose tolerance in an animal model of steatosis induced by high fat diet. PLoS ONE. 2013;8:e68626. 10.1371/journal.pone.0068626. 23861927 10.1371/journal.pone.0068626 PMC3702592

[61] Zhong H, Yu H, Chen J, Mok SWF, Tan X, Zhao B, et al. The short-chain fatty acid butyrate accelerates vascular calcification *via* regulation of histone deacetylases and NF-κB signaling. Vasc Pharmacol. 2022;146:107096. 10.1016/j.vph.2022.107096. 10.1016/j.vph.2022.107096 35952961

[62] Chakaroun RM, Olsson LM, Bäckhed F, Nature Publishing Group. The potential of tailoring the gut microbiome to prevent and treat cardiometabolic disease. Nat Rev Cardiol. 2023;20:217–35. 10.1038/s41569-022-00771-0. 36241728 10.1038/s41569-022-00771-0

[63] Tonch-Cerbu A-K, Boicean A-G, Stoia O-M, Teodoru M. Gut microbiota-derived metabolites in atherosclerosis: pathways, biomarkers, and targets. Int J Mol Sci. 2025;26:8488. 10.3390/ijms26178488. 40943409 10.3390/ijms26178488 PMC12429262

[64] Westerterp M, Berbée JFP, Pires NMM, van Mierlo GJD, Kleemann R, Romijn JA, et al. Apolipoprotein C-I is crucially involved in lipopolysaccharide-induced atherosclerosis development in apolipoprotein E-knockout mice. Circulation. 2007;116:2173–81. 10.1161/CIRCULATIONAHA.107.693382. 17967778 10.1161/CIRCULATIONAHA.107.693382

[65] Malik TH, Cortini A, Carassiti D, Boyle JJ, Haskard DO, Botto M. The alternative pathway is critical for pathogenic complement activation in endotoxin- and diet-induced atherosclerosis in low-density lipoprotein receptor-deficient mice. Circulation. 2010;122:1948–56. 10.1161/CIRCULATIONAHA.110.981365. 20974996 10.1161/CIRCULATIONAHA.110.981365 PMC2978131

[66] Serrano M, Moreno-Navarrete JM, Puig J, Moreno M, Guerra E, Ortega F, et al. Serum lipopolysaccharide-binding protein as a marker of atherosclerosis. Atherosclerosis. 2013;230:223–7. 10.1016/j.atherosclerosis.2013.07.004. 24075748 10.1016/j.atherosclerosis.2013.07.004

[67] Violi F, Cammisotto V, Bartimoccia S, Pignatelli P, Carnevale R, Nocella C, et al. Gut-derived low-grade endotoxaemia, atherothrombosis and cardiovascular disease. Nat Rev Cardiol. 2023;20:24–37. 10.1038/s41569-022-00737-2. 35840742 10.1038/s41569-022-00737-2 PMC9284488

[68] Zhu Y, Dwidar M, Nemet I, Buffa JA, Sangwan N, Li XS, et al. Two distinct gut microbial pathways contribute to meta-organismal production of phenylacetylglutamine with links to cardiovascular disease. Cell Host Microbe Elsevier. 2023;31:18-32.e9. 10.1016/j.chom.2022.11.015. 10.1016/j.chom.2022.11.015 PMC9839529 36549300

[69] Saha PP, Gogonea V, Sweet W, Mohan ML, Singh KD, Anderson JT, et al. Gut microbe-generated phenylacetylglutamine is an endogenous allosteric modulator of β2-adrenergic receptors. Nat Commun. 2024;15:6696. 10.1038/s41467-024-50855-3. 39107277 10.1038/s41467-024-50855-3 PMC11303761

[70] Agus A, Planchais J, Sokol H. Gut microbiota regulation of tryptophan metabolism in health and disease. Cell Host Microbe. 2018;23:716–24. 10.1016/j.chom.2018.05.003. 29902437 10.1016/j.chom.2018.05.003

[71] Qi Q, Li J, Yu B, Moon J-Y, Chai JC, Merino J, et al. Host and gut microbial tryptophan metabolism and type 2 diabetes: an integrative analysis of host genetics, diet, gut microbiome and circulating metabolites in cohort studies. Gut. 2022;71:1095–105. 10.1136/gutjnl-2021-324053. 34127525 10.1136/gutjnl-2021-324053 PMC8697256

[72] Roager HM, Licht TR. Microbial tryptophan catabolites in health and disease. Nat Commun. 2018;9:3294. 10.1038/s41467-018-05470-4. 30120222 10.1038/s41467-018-05470-4 PMC6098093

[73] Xue H, Chen X, Yu C, Deng Y, Zhang Y, Chen S, et al. Gut microbially produced indole-3-propionic acid inhibits atherosclerosis by promoting reverse cholesterol transport and its deficiency is causally related to atherosclerotic cardiovascular disease. Circ Res. 2022;131:404–20. 10.1161/CIRCRESAHA.122.321253. 35893593 10.1161/CIRCRESAHA.122.321253

[74] Koh A, Molinaro A, Ståhlman M, Khan MT, Schmidt C, Mannerås-Holm L, et al. Microbially produced imidazole propionate impairs insulin signaling through mTORC1. Cell. 2018;175:947-961.e17. 10.1016/j.cell.2018.09.055. 30401435 10.1016/j.cell.2018.09.055

[75] Mastrangelo A, Robles-Vera I, Mañanes D, Galán M, Femenía-Muiña M, Redondo-Urzainqui A, et al. Imidazole propionate is a driver and therapeutic target in atherosclerosis. Nature. 2025;645:254–61. 10.1038/s41586-025-09263-w. 40670786 10.1038/s41586-025-09263-w PMC12408353

[76] Nageswaran V, Carreras A, Reinshagen L, Beck KR, Steinfeldt J, Henricsson M, et al. Gut microbial metabolite imidazole propionate impairs endothelial cell function and promotes the development of atherosclerosis. Arterioscler Thromb Vasc Biol. 2025;45:823–39. 10.1161/ATVBAHA.124.322346. 40143816 10.1161/ATVBAHA.124.322346 PMC12017598

[77] Zhernakova A, Kurilshikov A, Bonder MJ, Tigchelaar EF, Schirmer M, Vatanen T, et al. Population-based metagenomics analysis reveals markers for gut microbiome composition and diversity. Science. 2016;352:565–9. 10.1126/science.aad3369. 27126040 10.1126/science.aad3369 PMC5240844

[78] Zhernakova DV, Le TH, Kurilshikov A, Atanasovska B, Bonder MJ, Sanna S, et al. Individual variations in cardiovascular-disease-related protein levels are driven by genetics and gut microbiome. Nat Genet. 2018;50:1524–32. 10.1038/s41588-018-0224-7. 30250126 10.1038/s41588-018-0224-7 PMC6241851

[79] Xu L, Zhang H, Wang Y, Yang A, Dong X, Gu L, et al. FABP4 activates the JAK2/STAT2 pathway via Rap1a in the homocysteine-induced macrophage inflammatory response in ApoE−/− mice atherosclerosis. Lab Invest Nature Pub Group. 2022;102:25–37. 10.1038/s41374-021-00679-2. 10.1038/s41374-021-00679-2 PMC8695379 34725437

[80] Kim YS, Unno T, Kim B-Y, Park M-S. Sex differences in gut microbiota. World J Mens Health. 2020;38:48–60. 10.5534/wjmh.190009. 30929328 10.5534/wjmh.190009 PMC6920072

[81] Sharma A, Kapur S, Kancharla P, Yang T. Sex differences in gut microbiota, hypertension, and cardiovascular risk. Eur J Pharmacol. 2025;987:177183. 10.1016/j.ejphar.2024.177183. 39647571 10.1016/j.ejphar.2024.177183 PMC11714433

[82] Yoon K, Kim N. Roles of sex hormones and gender in the gut microbiota. J Neurogastroenterol Motil. 2021;27:314–25. 10.5056/jnm20208. 33762473 10.5056/jnm20208 PMC8266488

[83] Mayneris-Perxachs J, Arnoriaga-Rodríguez M, Luque-Córdoba D, Priego-Capote F, Pérez-Brocal V, Moya A, et al. Gut microbiota steroid sexual dimorphism and its impact on gonadal steroids: influences of obesity and menopausal status. Microbiome. 2020;8:136. 10.1186/s40168-020-00913-x. 32951609 10.1186/s40168-020-00913-x PMC7504665

[84] Zhou J, Liu L, Wu P, Zhao L, Wu Y. *Fusobacterium nucleatum* accelerates atherosclerosis via macrophage-driven aberrant proinflammatory response and lipid metabolism. Front Microbiol. 2022. 10.3389/fmicb.2022.798685. 35359716 10.3389/fmicb.2022.798685 PMC8963492

[85] Bradley E, Haran J. The human gut microbiome and aging. Gut Microbes. 2024;16:2359677. 10.1080/19490976.2024.2359677. 38831607 10.1080/19490976.2024.2359677 PMC11152108

[86] Ghosh TS, Rampelli S, Jeffery IB, Santoro A, Neto M, Capri M, et al. Mediterranean diet intervention alters the gut microbiome in older people reducing frailty and improving health status: the NU-AGE 1-year dietary intervention across five European countries. Gut. 2020;69:1218–28. 10.1136/gutjnl-2019-319654. 32066625 10.1136/gutjnl-2019-319654 PMC7306987

[87] Kim ES, Yoon BH, Lee SM, Choi M, Kim EH, Lee B-W, et al. Fecal microbiota transplantation ameliorates atherosclerosis in mice with C1q/TNF-related protein 9 genetic deficiency. Exp Mol Med. 2022;54:103–14. 10.1038/s12276-022-00728-w. 35115674 10.1038/s12276-022-00728-w PMC8894390

[88] Wang Z, Roberts AB, Buffa JA, Levison BS, Zhu W, Org E, et al. Non-lethal inhibition of gut microbial trimethylamine production for the treatment of atherosclerosis. Cell. 2015;163:1585–95. 10.1016/j.cell.2015.11.055. 26687352 10.1016/j.cell.2015.11.055 PMC4871610

[89] Cao H, Zhu Y, Hu G, Zhang Q, Zheng L. Gut microbiome and metabolites, the future direction of diagnosis and treatment of atherosclerosis? Pharmacol Res. 2023;187:106586. 10.1016/j.phrs.2022.106586. 36460280 10.1016/j.phrs.2022.106586

